# Integrated Management of Cardio-Renal-Metabolic Disorders in India: A Nationwide Physician Survey

**DOI:** 10.7759/cureus.105480

**Published:** 2026-03-19

**Authors:** Abdul Hamid Zargar, Johann Christopher, Sanjeev Hiremath, Sameer Muchhala, Vishal Gala

**Affiliations:** 1 Endocrinology, Center for Diabetes and Endocrine Care, Srinagar, IND; 2 Cardiology, Care Hospitals, Hyderabad, IND; 3 Internal Medicine, Sagar Hospitals, Bangalore, IND; 4 Medical Affairs, Zydus Lifesciences Limited, Mumbai, IND; 5 Medical Affairs, Zydus Healthcare Limited, Mumbai, IND

**Keywords:** cardiovascular disease, cardiovascular renal metabolic (careme) disorders, chronic kidney disease, diabetes, multimorbidity

## Abstract

Background

Cardiovascular, renal, and metabolic (CaReMe) disorders represent an interlinked group of conditions. Multimorbidity is frequent among patients with diabetes; 43% also have hypertension, and nearly 28% of hypertensive individuals have diabetes. Despite growing evidence and guideline recommendations for integrated care, limited data exist on how physicians in India perceive and manage CaReMe conditions in routine practice.

Objectives

To describe physician-reported practices, diagnostic routines, perceptions, and barriers related to the integrated management of cardio-renal-metabolic (CaReMe) conditions among practicing physicians in India using a cross-sectional survey design.

Methods

A descriptive survey was conducted among 1,025 physicians, including consulting physicians, diabetologists, cardiologists, endocrinologists, and nephrologists, with at least one year of clinical experience. Data were collected using a pre-tested, structured electronic questionnaire and analysed descriptively.

Results

The majority of respondents were consulting physicians (57.7%, n = 591) or diabetologists (25.7%, n = 263). The majority had 11-20 years of clinical experience, and one-third reported seeing more than 200 patients per month. According to the respondents, the most common comorbid conditions were diabetes (75.8%, n = 777), dyslipidaemia (74.1%, n = 760), obesity (71.1%, n = 729), and hypertension (70.4%, n = 721). The most frequently performed routine diagnostic assessments were glycated haemoglobin (HbA1c; 82.4%, n = 845), lipid profile (80.7%, n = 827), estimated glomerular filtration rate (eGFR) (78.0%, n = 800), and microalbuminuria (74.8%, n = 767). However, only 16.1% (n = 165) of physicians recognised CaReMe conditions as direct contributors to cardiovascular and renal outcomes, while 82% (n = 841) considered integrated management to be important. The most common barriers included patient affordability (76%, n = 779), lack of awareness (74.1%, n = 760), limited consultation time (69.7%, n = 714), and inadequate guidelines (63.1%, n = 647). Across all three disease-combination categories, the largest proportion of physicians selected the five to 10 patients-per-month category for newly diagnosed cases.

Conclusion

This nationwide survey highlights the high adoption of recommended diagnostic practices but reveals a significant disconnect between the implementation of diagnostic practices and physician awareness of the interconnections between CaReMe. Persistent barriers, including affordability, the absence of standardized national guidelines, and limited consultation time, underscore the urgent need for coordinated policy and educational interventions. Strengthening physician training, harmonizing clinical guidelines, improving financial accessibility, and promoting holistic care models are essential steps toward improving patient outcomes and mitigating the growing CaReMe burden in India.

## Introduction

Cardiovascular, renal, and metabolic (CaReMe) disorders represent a converging epidemic that is reshaping the profile of non-communicable diseases worldwide. In India, nationally representative data show that hypertension affects approximately 30.3% of adult men and 28.6% of women, while diabetes is present in 19.7% of men and 17.4% of women. Multimorbidity is frequent among patients with diabetes; 43% also have hypertension, and nearly 28% of hypertensive individuals have diabetes [1]. Chronic kidney disease (CKD) further complicates this landscape. The Screening and Early Evaluation of Kidney Disease (SEEK)-India cohort reported a CKD prevalence of 17.2%, with about 6% of participants having stage 3 or worse disease [2].

Worldwide, there is a growing recognition that cardiovascular, renal, and metabolic conditions are interconnected at the pathophysiological level. Shared mechanisms, such as chronic inflammation, oxidative stress, hyperglycaemia, activation of the renin-angiotensin-aldosterone system, and neurohormonal dysfunction, create a vicious cycle in which dysfunction in one organ accelerates deterioration in others [3]. The 2017 Global Burden of Disease Study documented a substantial increase in the global burden of chronic kidney disease since 1990 and underscored its major contribution to morbidity, mortality, and cardiovascular risk [4]. At the same time, projections indicate that by 2030, overweight and obesity will affect nearly three billion adults, about half of the world’s population, while the number of people with diabetes is expected to rise from 529 million in 2021 to 783 million by 2045 [5]. Together, these data highlight the escalating burden of CaReMe multimorbidity, which drives excess morbidity and mortality while straining health systems.

To address this overlap, clinical practice guidelines are increasingly advocating for coordinated management strategies. Evidence-based therapies, such as renin-angiotensin-aldosterone system inhibitors, sodium-glucose cotransporter-2 inhibitors, and glucagon-like peptide-1 receptor agonists, have demonstrated benefits across cardiovascular, renal, and metabolic outcomes. Observational analyses of cardiovascular outcome trials reveal that drugs initially developed for diabetes or cardiovascular disease often confer protection across multiple organ systems. This cross-organ efficacy has driven multispecialty consensus statements that encourage clinicians to move beyond siloed approaches and adopt holistic care frameworks [6]. The American Heart Association (AHA) recently issued 10 takeaways for cardiovascular-kidney-metabolic health, emphasising strategies to reduce care fragmentation, integrate screening for social determinants of health, and prioritise lifestyle modification and weight loss. Their advisory further provides algorithms for selecting cardioprotective antihyperglycemic agents and recommends measuring the urine albumin-creatinine ratio alongside the estimated glomerular filtration rate for comprehensive risk assessment [7]. From a European perspective, Italian experts recommend early detection of hypertension, waist circumference, glycated haemoglobin, lipid profile, and renal markers at the primary care level, supported by seamless referral pathways and collaboration among cardiologists, nephrologists, and diabetologists. Despite these advances, implementation remains inconsistent due to workforce shortages, limited guidance on multimorbidity, and inadequate access to diagnostic and therapeutic resources [8].

Alongside pharmacological innovations, lifestyle interventions play a pivotal role in CaReMe management. A meta-analysis of over 679,000 participants reported that adherence to a Mediterranean diet rich in plant-based foods was associated with a 23% reduction in all-cause mortality and a 27% reduction in cardiovascular mortality [9]. However, dietary patterns vary considerably across regions, and recommendations must be contextualised accordingly. In India, traditional diets differ substantially between northern and southern states, with variations in cereal staples, cooking oils, and the proportion of plant-based versus animal-derived foods. These regional dietary differences may differentially influence cardiometabolic risk profiles, underscoring the need for culturally tailored nutritional guidance rather than uniform adoption of Western dietary models. Regular physical activity has been shown to lower risks of metabolic syndrome, cardiovascular disease, and type 2 diabetes, with international guidelines recommending at least 150 minutes per week of moderate to vigorous aerobic exercise supplemented by resistance training [10]. These strategies complement pharmacotherapy by addressing upstream risk factors and align with the AHA advisory’s emphasis on tackling excess adiposity and social determinants of health [7]. Nonetheless, real-world adoption is uneven: younger, lower-risk patients are more likely to receive newer cardioprotective agents than those with the highest cardiorenal risk [11]. At the same time, healthcare systems continue to operate in fragmented silos [8].

Amidst increasing multimorbidity, changing guidelines, and implementation challenges, there is limited understanding of how Indian physicians perceive and handle CaReMe conditions in everyday practice. To address this, a survey of 1,025 consulting physicians, diabetologists, cardiologists, endocrinologists, and nephrologists across India was conducted. The survey explored clinical practices, diagnostic routines, attitudes toward integrated care, and perceived barriers. By capturing these physician-reported patterns and challenges, our study provides insights into opportunities for strengthening integrated care pathways, tailoring educational initiatives, and aligning real-world practice with emerging evidence on holistic CaReMe management.

## Materials and methods

Study design and setting

This was a descriptive, cross-sectional survey conducted among physicians involved in the management of cardiovascular, renal, and metabolic (CaReMe) conditions. The survey was conducted across diverse regions of India to capture variation in clinical practices and perspectives. Participants were recruited using a convenience sampling approach through professional medical networks and electronic outreach across four geographical zones of India (North, South, East, and West). No formal sample size calculation was performed; however, the target was to obtain a geographically and specialty-diverse sample of practising physicians managing CaReMe conditions. The survey was conducted between February 2025 and November 2025.

Study population

A total of 1,025 physicians completed the survey and were included in the final analysis. Eligible participants comprised consulting physicians, cardiologists, endocrinologists, nephrologists, and diabetologists with a minimum of one year of independent clinical practice. Physicians not actively engaged in patient care or those providing incomplete or invalid responses were excluded.

Study objective

Primary Objective

To describe physician-reported practices related to the management of cardio-renal-metabolic (CaReMe) conditions among practicing physicians in India using a cross-sectional survey.

Specific Objectives

To assess physician-reported diagnostic practices used for evaluating CaReMe conditions. To evaluate physicians’ perceptions regarding the burden and integrated management of CaReMe disorders. To identify barriers faced by physicians in implementing multidisciplinary management of CaReMe conditions in routine clinical practice.

Data collection procedures

Data were collected using a structured, pre-tested, self-administered electronic questionnaire. The questionnaire was developed by the study team based on a review of existing literature on CaReMe multimorbidity and clinical practice patterns and was reviewed for content validity by subject-matter experts prior to administration. The instrument comprised eight closed-ended items organised into four domains: (a) physician demographics, including specialisation, years of clinical practice, region of practice, and average monthly patient volume; (b) clinical burden, including commonly observed comorbidities (Question 1), estimated prevalence of cardiovascular, renal, and metabolic conditions among patients (Question 6), overlap of specific comorbidity combinations (Question 7), and frequency of newly diagnosed multimorbid patients per month (Question 8); (c) diagnostic practices, including routine assessments performed for CaReMe patients (Question 5); and (d) physician perceptions and barriers, including recognition of CaReMe contributions to cardiovascular and renal outcomes (Question 2), perceived importance of integrated management (Question 3), and barriers to adopting a multidisciplinary approach (Question 4). Questions 1, 4, and 5 permitted multiple responses. Questions 6 and 7 collected physician-estimated percentages as continuous variables. Question 8 used ordinal categories (<5, 5-10, 11-20, >20) to capture the monthly frequency of newly diagnosed patients with overlapping CaReMe conditions (Appendix).

Eligible physicians were identified through professional medical databases and invited to participate via personalised electronic communication (e-mail or secure messaging). Each invitee received a unique link granting access to the online survey platform. The questionnaire was accessible for a defined data-collection window, during which a single reminder was sent to non-respondents. Participation was voluntary, and no financial or other incentives were provided. The questionnaire included mandatory-field validation to minimise incomplete submissions; responses with missing core data were excluded at the data-cleaning stage. As a cross-sectional survey, the study did not involve follow-up visits or longitudinal assessments.

Sampling method

Physicians were recruited using a convenience sampling approach through professional medical networks, electronic communication platforms, and physician outreach channels. Participation was voluntary and limited to practicing physicians in India with at least one year of clinical experience. The survey targeted consulting physicians, diabetologists, cardiologists, endocrinologists, and nephrologists involved in the management of cardio-renal-metabolic conditions.

Questionnaire development and data collection

A structured electronic questionnaire was developed following a review of the literature and expert input from clinicians experienced in CaReMe disease management. The draft questionnaire was reviewed by subject-matter experts to assess content validity, clarity, and relevance, and minor revisions were made before dissemination. The final instrument collected information on physician demographics, clinical practice patterns, diagnostic routines, perceptions regarding integrated CaReMe management, and barriers to multidisciplinary care.

Question 8 used ordinal response categories (<5, 5-10, 11-20, >20) to capture the monthly frequency of newly diagnosed patients with overlapping CaReMe conditions reported by physicians. This item measured physician-reported frequency of newly diagnosed cases per month and did not assess time to diagnosis or diagnostic delay.

Statistical analysis

All analyses were descriptive. Categorical variables were summarised as frequencies and percentages. Continuous variables derived from physician-estimated prevalence data (Questions 6 and 7) were expressed as mean ± standard deviation when normally distributed, or as median with interquartile range otherwise. No inferential statistical tests or subgroup comparisons were performed, consistent with the exploratory, descriptive design of the survey. Data were cleaned, coded, and analysed using standard statistical software. Multiple-response items were analysed on a per-option basis, with the denominator being the total number of respondents (N = 1,025).

Ethical considerations

In accordance with applicable national ethical guidance, including Indian Council of Medical Research (ICMR) principles for low-risk observational research, the study was deemed exempt from formal Institutional Ethics Committee review because of its non-interventional design and use of fully anonymized survey data. Documentation of this exemption/deemed-not-required determination was obtained prior to data collection. Electronic informed consent was obtained from all participants.

## Results

A total of 1,025 physicians across India participated in the survey. Among them, consulting physicians formed the largest group (591; 57.7%), followed by diabetologists (263; 25.7%), cardiologists (43; 4.2%), endocrinologists (35; 3.4%), and nephrologists (10; 1.0%); 83 (8.1%) belonged to other specialties (Table 1). More than half of the respondents (57.3%) had 11-20 years of clinical experience, while 22.7% had five to 10 years, 12% had less than five years, and 8.6% had more than 20 years of experience.

**Table 1:** Participant characteristics. Note: Percentages are based on total respondents (N = 1,025).

Variable	Frequency	Percentage
Specialization		
Consulting Physician	591	57.7%
Diabetologist	263	25.7%
Cardiologist	43	4.2%
Endocrinologist	35	3.4%
Nephrologist	10	1.0%
Other	83	8.1%
Years of clinical practice		
<5 years	118	11.5%
5-10 years	233	22.7%
11-20 years	587	57.3%
>20 years	87	8.6%
Region		
East	524	51.1%
West	255	24.9%
South	163	15.9%
North	83	8.1%
Patients per month		
<50	203	19.8%
50-100	321	31.3%
101-200	163	15.9%
>200	338	33.0%

Participants represented all major regions of India, with the eastern region contributing the highest proportion (51.1%), followed by the western (24.9%), southern (15.9%), and northern (8.1%) regions. Regarding patient volume, 33% of physicians reported consulting more than 200 patients per month, 31.3% saw 50-100 patients, 15.9% saw 101-200 patients, and 19.8% saw fewer than 50 patients per month (Table 1).

Patient comorbidity profile

According to respondents, the mean percentage of patients with cardiovascular diseases such as coronary artery disease and heart failure was 25.13%, indicating a significant prevalence of cardiac issues in the group. Kidney involvement, including chronic kidney disease and albuminuria, had a median prevalence of 20%. Metabolic disorders like diabetes mellitus, dyslipidaemia, and obesity had the highest median prevalence at 38.44%.

**Figure 1:**
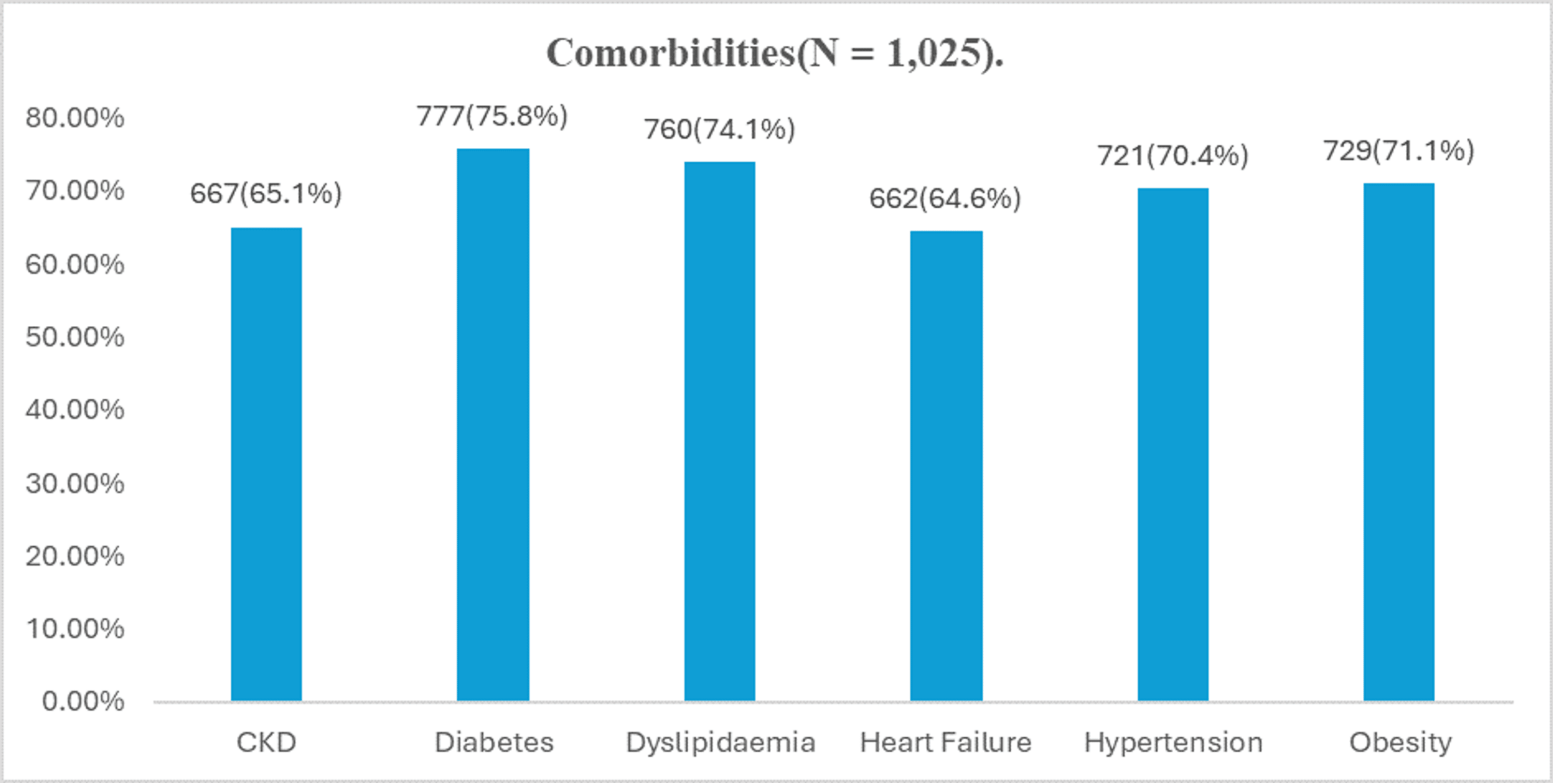
Prevalence of comorbidities among patients (N = 1,025). CKD: chronic kidney disease.

Comorbidities were very common among CaReMe patients. The most frequently reported were diabetes (75.8%), dyslipidaemia (74.1%), and obesity (71.1%), followed by hypertension (70.4%). Cardiovascular and renal conditions were reported by over 60% of respondents as common comorbidities in their patients (Figure 1). According to physician estimates, 71.8% of patients had more than two comorbid conditions, 6.24% had two, and 21.95% had a single comorbid condition.

Additionally, the comorbidity assessment showed a complex overlap of conditions typical of advanced cardiometabolic syndrome. Among patients, 20% had both diabetes and cardiovascular disease, while 15% had diabetes and chronic kidney disease. Additionally, 10% experienced co-occurring cardiovascular and renal diseases, and another 10% had the triad of diabetes, cardiovascular disease, and chronic kidney disease, indicating a significant clustering of these interrelated disorders. The metabolic overlap was even more evident, with 30% of patients presenting both diabetes and dyslipidaemia, and another 30% having the combined burden of diabetes, dyslipidaemia, and hypertension.

Diagnostic assessments

**Figure 2:**
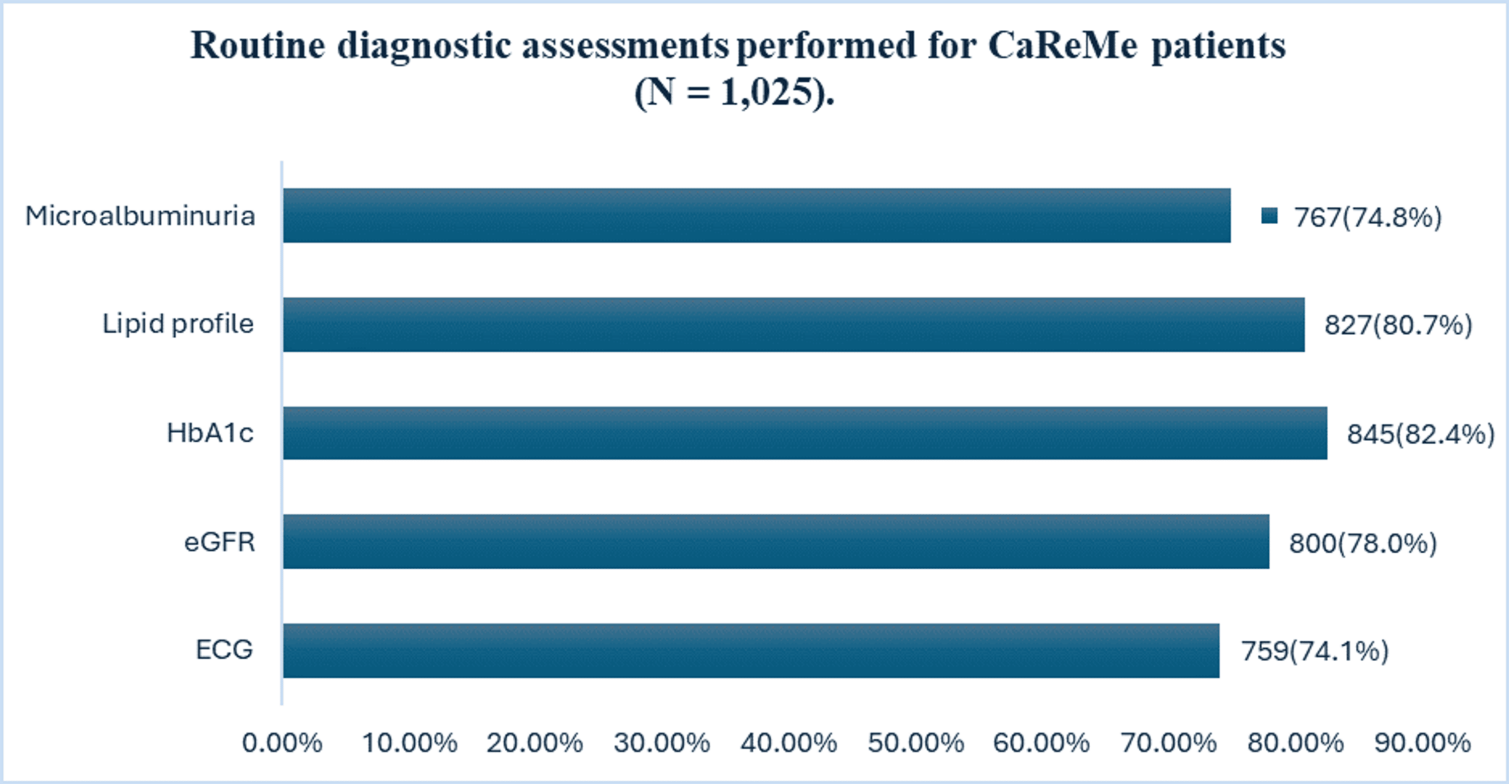
Routine diagnostic assessments performed for CaReMe patients (N = 1,025). CaReMe: cardiovascular, renal, and metabolic; HbA1c: glycated haemoglobin; eGFR: estimated glomerular filtration rate.

Routine diagnostic assessments were widely adopted. According to the respondents, glycated haemoglobin (HbA1c) (82.4%), lipid profile (80.7%), and estimated glomerular filtration rate (eGFR) (78.0%) were the most frequently performed tests, followed by microalbuminuria (74.8%) and ECG (74.1%) (Figure 2).

Perceptions of CaReMe disease burden and integrated care

**Figure 3:**
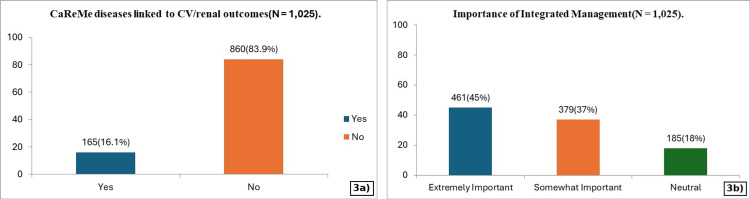
Perceptions of disease burden and integrated care (N = 1,025). (a) Physician recognition of CaReMe conditions as direct contributors to cardiovascular and renal outcomes; and (b) physician perception regarding the importance of integrated management of CaReMe conditions. CaReMe: cardiovascular, renal, and metabolic; CV: cardiovascular.

A lesser proportion of physicians (16.1%) recognised that CaReMe diseases could directly contribute to cardiovascular/renal outcomes and mortality. Nevertheless, 45% believed that managing these conditions in an integrated manner was “extremely important,” while 37% considered it “somewhat important” (Figure 3).

Barriers to multidisciplinary management

**Figure 4:**
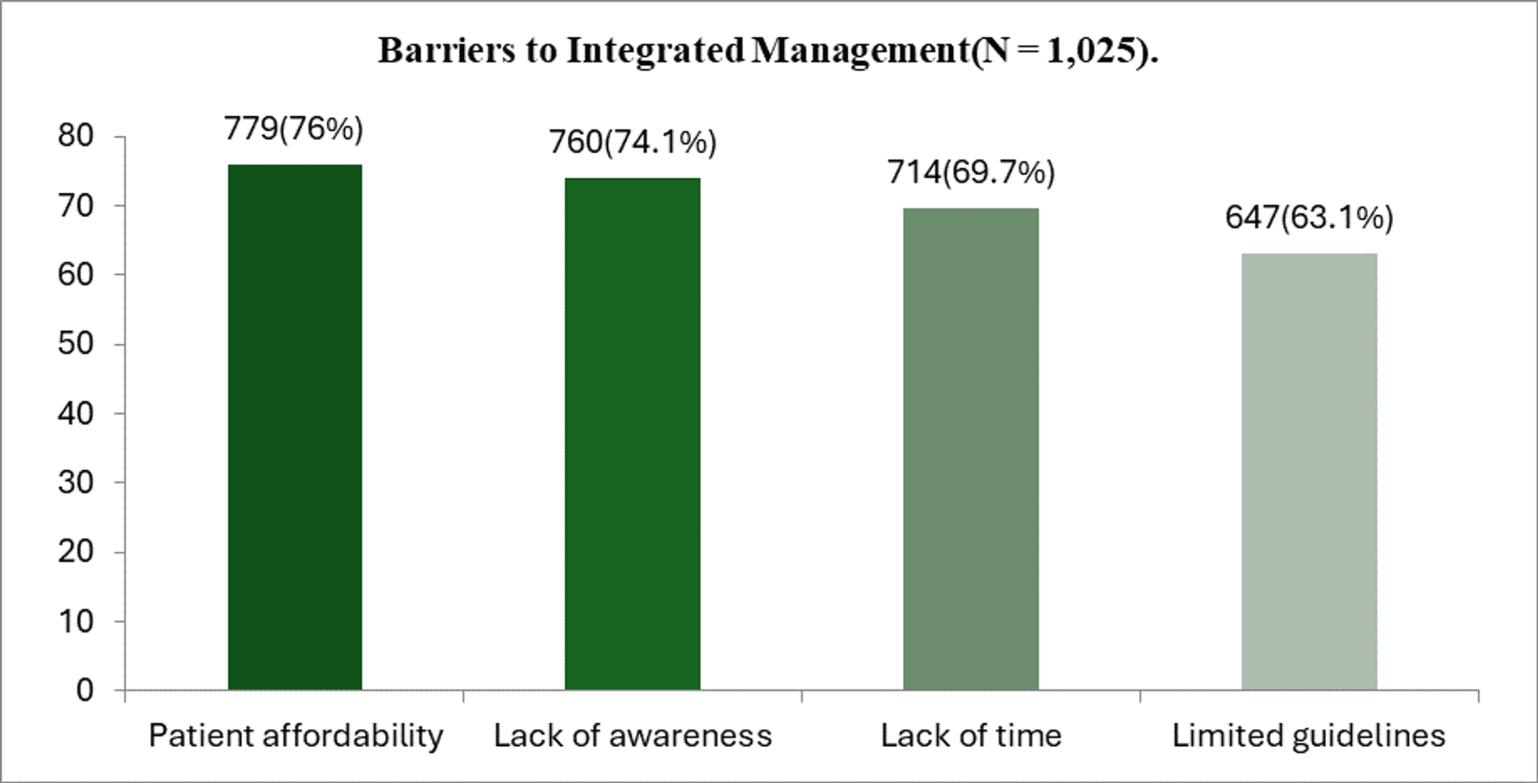
Barriers to multidisciplinary management (N = 1,025).

The most common barriers to multidisciplinary care were patient affordability (76%), followed by a lack of awareness (74.1%) and a lack of time (69.7%). Limited availability of guidelines was also reported by 63.1% of respondents (Figure 4).

Across all three disease-combination categories, the largest proportion of physicians selected the five to 10 patients-per-month category for newly diagnosed cases. For metabolic + cardiovascular disease, 44.8% of physicians reported seeing five to 10 such newly diagnosed patients per month. Corresponding proportions were 42.2% for cardiovascular + renal disease and 34.3% for metabolic + cardiovascular + renal disease (Table 2).

**Table 2:** Monthly frequency distribution of newly diagnosed patients with multiple CaReMe conditions (physician-reported categories) (N = 1,025). CaReMe: cardiovascular, renal, and metabolic; CVD: cardiovascular disease.

Category	<5 patients/month	5-10 patients/month	11-20 patients/month	>20 patients/month
Metabolic + CVD	113 (11.0%)	459 (44.8%)	191 (18.6%)	262 (25.6%)
CVD + Renal	262 (25.6%)	433 (42.2%)	193 (18.8%)	137 (13.4%)
Metabolic + CVD + Renal	252 (24.6%)	351 (34.3%)	189 (18.4%)	233 (22.7%)

## Discussion

This nationwide cross-sectional survey provides insight into how Indian physicians perceive and manage cardiovascular, renal, and metabolic (CaReMe) disorders. The findings indicate a substantial burden of multimorbidity in routine clinical practice and widespread use of key diagnostic investigations such as HbA1c, lipid profiles, and renal markers. Despite this diagnostic engagement, physician recognition of the direct link between CaReMe conditions and adverse cardiovascular and renal outcomes remains limited. Although most respondents acknowledged the importance of integrated management, barriers, including affordability, limited awareness, the absence of harmonised guidelines, and time constraints, continue to hinder the implementation of multidisciplinary care in India [12,13].

The survey findings are consistent with broader epidemiological evidence from India. Diabetes, dyslipidaemia, obesity, and hypertension were reported as the most frequent comorbidities, in line with population-based studies. For example, a community-based cross-sectional study from the Etawah district of Uttar Pradesh reported a substantial burden of cardiometabolic risk factors, with approximately 38%-39% of adults overweight or obese, 29%-36% having hypertension, 4%-8% having diabetes, and around 27% having dyslipidaemia [14]. Similarly, the Indian Council of Medical Research-India Diabetes study (ICMR-INDIAB) survey documented a high prevalence of dyslipidaemia in Indian adults, with at least one lipid abnormality present in nearly 79% of participants, low high-density lipoprotein (HDL) cholesterol affecting more than 72%, and hypertriglyceridaemia present in approximately 30% [15]. Multimorbidity has also been observed in electronic medical record cohorts, where the coexistence of hypertension, diabetes, and dyslipidaemia was reported in more than 6% of patients [16]. These findings highlight the substantial burden of CaReMe conditions in India and align with the reports from our respondents. The high prevalence of metabolic disorders and kidney involvement suggests that metabolic risk factors and renal abnormalities are key components of the cardio-renal-metabolic disease spectrum in routine clinical practice. The observed overlap of diabetes, cardiovascular disease, and chronic kidney disease further reflects the close interrelationship between metabolic control, vascular health, and renal dysfunction, identifying a high-risk population that may benefit from integrated management strategies. Encouragingly, most physicians reported routinely performing investigations such as HbA1c, lipid profiles, estimated glomerular filtration rate, microalbuminuria, and electrocardiography, which are recommended by integrated cardiovascular-renal-metabolic screening frameworks [17].

However, recognition of the syndromic nature of CaReMe remained limited, with only 16.1% of physicians acknowledging the direct link between these interconnected conditions and adverse cardiovascular or renal outcomes. The low proportion of physicians explicitly recognising CaReMe conditions as direct contributors to cardiovascular and renal outcomes, despite frequent use of relevant diagnostic tests, may reflect a gap between disease-specific clinical vigilance and conceptual adoption of an integrated CaReMe framework. In other words, physicians may routinely screen for individual metabolic, renal, and cardiovascular abnormalities without necessarily labelling them as components of a unified interlinked syndrome. This disconnect, despite widespread diagnostic activity, suggests a knowledge-practice gap that may be influenced by factors such as speciality, years of experience, or patient volume. The distribution of physician responses indicates that newly diagnosed cases of CaReMe multimorbidity are encountered regularly in routine clinical practice. This knowledge gap is consistent with international literature, which cites limited awareness of chronic kidney disease and a lack of familiarity with guideline-directed therapies among clinicians [18].

In addition to gaps in awareness, systemic challenges emerged as major obstacles to integrated management. Affordability was the most commonly cited barrier, reflecting the high out-of-pocket expenditure associated with chronic disease management in India and underscoring the importance of cost-effective integrated care models [19,20]. Lack of awareness, absence of harmonised guidelines, and limited consultation time were also prominent, consistent with global evidence on barriers to multidisciplinary care [21]. The survey further revealed diagnostic delays, with many multimorbid patients being diagnosed five to 10 months after initial presentation. This is particularly concerning given the asymptomatic nature of early cardiovascular-kidney-metabolic (CKM) disease, where opportunities for early intervention are often missed. International recommendations emphasise routine assessment of blood pressure, creatinine, urinary albumin-to-creatinine ratio, and glycated haemoglobin for timely detection [22], yet the realities of limited resources, high patient loads, and administrative constraints may explain these diagnostic delays. Patient-level factors such as low health literacy and financial constraints may further contribute to underdiagnosis and delayed care-seeking.

Indian physicians demonstrate a strong awareness of the burden posed by cardiovascular, renal, and metabolic (CaReMe) multimorbidity, as evidenced by the widespread use of recommended diagnostic assessments. Yet, the absence of a unifying conceptual framework continues to impede the transition from diagnostic practices to fully integrated, multidisciplinary care. Embedding the CaReMe construct into undergraduate medical education and structured continuing professional development could enhance understanding of the shared pathophysiology linking cardiovascular, renal, and metabolic disorders. In particular, dedicated continuing medical education (CME) programmes focused on the evolving CaReMe paradigm are needed to update practising physicians on recent advances in integrated cardio-renal-metabolic care, including newer therapeutic agents with cross-organ benefits and risk-stratification algorithms. The formulation of harmonised national guidelines, bridging cardiology, nephrology, endocrinology, and primary care, would further reduce fragmentation and promote coordinated management strategies.

This study has several limitations that should be considered when interpreting the findings. First, the survey used a convenience sampling approach, which may introduce selection bias and limits the ability to generalise the results to all physicians in India. Although participants were recruited from multiple regions across the country, the survey should be interpreted as nationwide in geographic scope rather than nationally representative in a probabilistic sense. Second, the findings are based on self-reported physician responses, which may be influenced by recall bias or social desirability bias. Third, because the currently available dataset consists of aggregate survey outputs, subgroup analyses based on physician specialty, years of experience, patient volume, or regional practice patterns were not performed. Such analyses could provide additional insights and should be explored in future research using respondent-level datasets.

Addressing economic barriers remains equally critical. Expansion of insurance coverage and enforcement of rational drug pricing policies are necessary to improve access to comprehensive care. In parallel, restructuring service delivery through team-based models, including task-sharing with allied health professionals, may alleviate time constraints and support more efficient care delivery in high-volume clinical environments [23,24]. The cross-sectional and self-reported design of the dataset may introduce recall and reporting biases, while the voluntary nature of participation increases the likelihood of selection effects, particularly from urban-based physicians. Because the present report is based on aggregate descriptive survey outputs, subgroup analyses by speciality, years of experience, patient volume, and region were not performed in this manuscript. These analyses would be valuable in future work to determine whether the observed awareness-practice patterns vary across physician groups and practice settings. The lack of inferential statistical analysis also limits the exploration of subgroup variations in clinical practice. Nevertheless, the findings offer meaningful insight into prevailing physician behaviours and systemic challenges, forming a credible foundation for future educational, policy, and health system interventions aimed at enhancing integrated management of cardio-renal-metabolic conditions.

## Conclusions

Indian physicians report a significant burden of cardiovascular, renal, and metabolic (CaReMe) multimorbidity in clinical practice and demonstrate widespread adoption of recommended diagnostic strategies. Despite this, critical gaps remain in recognising integrated disease pathways, compounded by persistent barriers such as affordability, the lack of harmonised clinical guidelines, and limited opportunities for multidisciplinary collaboration. While interpretation should stay cautious due to the cross-sectional design, self-reported data, and potential for selection bias, the findings offer a timely and realistic view of care delivery patterns in India. Incorporating the CaReMe framework into undergraduate medical education and ongoing professional development could improve clinicians' understanding of shared disease mechanisms. Structured continuing medical education (CME) programmes should be prioritised to disseminate the updated CaReMe concept among practising clinicians, with attention to regional variations in dietary patterns and disease profiles. Developing unified national guidelines that connect cardiology, nephrology, endocrinology, and primary care might reduce care fragmentation and encourage coordinated, patient-centred management. Complementary policy actions, such as expanding insurance coverage, rationalising drug prices, and establishing team-based care models with nurse-led and allied health task-sharing, are equally important to tackle systemic challenges. Collectively, these approaches can help advance integrated care and better outcomes for patients with cardio-renal-metabolic disease in India. Future studies using probability-based sampling or respondent-level datasets could further explore variations in physician practices by specialty, years of experience, patient volume, and regional healthcare contexts.

## Appendices

Integrated Management of Cardio-Renal-Metabolic

Disorders in India

A Nationwide Physician Survey

CaReMe Disease Questionnaire

Section A: Physician Demographics

Field

Options (Please select)

Specialization

□ Consulting Physician

□ Cardiologist

□ Endocrinologist

□ Nephrologist

□ Diabetologist

□ Other (Specify): _______________

Years of Clinical Practice

□ < 5 years

□ 5-10 years

□ 11-20 years

□ > 20 years

Region of Practice

□ North

□ South

□ East

□ West

Average CaReMe Patients per Month

□ < 50

□ 50-100

□ 101-200

□ > 200

Section B: Clinical Observations

1. Which of the following conditions are most commonly observed as part of CaReMe disease in your clinical practice?

(Select all that apply)

□ Diabetes

□ Dyslipidemia

□ Obesity

□ Hypertension

□ CKD

□ Heart Failure

□ Others: _______________

2. Do you think that CaReMe diseases can lead to cardiovascular and/or renal outcomes and mortality?

□ A.  Yes

□ B.  No

3. How important is it to manage CaReMe diseases in an integrated manner?

□ Extremely important

□ Somewhat important

□ Neutral

□ Not important

4. What barriers do you face in adopting a multidisciplinary approach for CaReMe disease management?

(Select all that apply)

□ Lack of time

□ Limited guidelines

□ Lack of awareness

□ Patient affordability

□ Other (Specify): _______________

Section C: Diagnostic & Management Practices

5. Which of the following diagnostic assessments do you routinely perform for CaReMe patients?

(Select all that apply)

□ Lipid profile

□ HbA1c

□ eGFR

□ ECG

□ Microalbuminuria

□ Others (Specify): _______________

Section D: Patient Burden & Disease Overlap

6. What percentage of your total patients have the following conditions?

(Approximate percentages)

Condition

Approximate %

Cardiovascular disease (CAD, heart failure, etc.)

_______  %

Renal disease (CKD, albuminuria, etc.)

_______  %

Metabolic disorders (diabetes, dyslipidemia, obesity)

_______  %

7. What percentage of your total patient population falls under the following categories?

(Approximate percentages - Two or more interconnected CaReMe conditions)

Condition Combination

Approximate %

Diabetes + CVD

_______  %

Diabetes + CKD

_______  %

CVD + CKD

_______  %

Diabetes + CVD + CKD

_______  %

Diabetes + Dyslipidemia

_______  %

Diabetes + Dyslipidemia + Hypertension

_______  %

Others (Please specify): _______________

_______  %

8. How frequently do you see newly diagnosed cases involving multiple CaReMe conditions in a typical month?

Patients who are newly diagnosed with metabolic and cardiovascular disease per month :

<5

5-10

11-20

>20

Patients who are newly diagnosed with cardiovascular and renal disease per month :

<5

5-10

11-20

>20

Patients who are newly diagnosed with metabolic, cardiovascular, and renal disease per month (all three):

<5

5-10

11-20

>20

Thank you for participating in this nationwide physician survey. Your responses are confidential and will be used solely for research purposes.
